# Short-term triphenyltin exposure alters microbial homeostasis in the silkworm (*Bombyx mori*) midgut

**DOI:** 10.1038/s41598-023-41948-y

**Published:** 2023-09-13

**Authors:** Wenlin Zhou, Xing Zhang, Xuedong Chen, Xuehui Wu, Aihong Ye, Jinru Cao, Xiaolong Hu

**Affiliations:** 1 Institute of Sericulture and Tea, Zhejiang Academy of Agricultural Sciences, Hangzhou, 310021 China; 2 School of Chemistry and Life Science, Suzhou University of Science and Technology, Suzhou, 215009 China; 3 School of Biology & Basic Medical Science, Soochow University, Suzhou, 215123 China

**Keywords:** Computational biology and bioinformatics, Microbiology, Environmental sciences

## Abstract

Triphenyltin (TPT) is a widespread synthetic chemical used in many fields and its potential risk to organisms has been comprehensively investigated using different animal models and species. Currently, little is known about the effects of TPT exposure on microbial midgut diversity, therefore we explored these effects in the lepidopterous silkworm model using 16S rDNA sequencing. In total, 5273 and 5065 operational taxonomic units (OTUs) were identified in control and TPT-exposure group samples, ranging from 424 to 728 OTUs/sample. Alpha-diversity analyses revealed that TPT exposure induced the fluctuations of gut microbial diversity and abundance while beta-diversity analyses identified a distinct impact on major gut microbiota components. In our microbiome analyses, 23 phyla and 353 genera were recognized in the control group, while 20 phyla and 358 genera were recognized in the TPT exposure group. At the genus level, midgut microbiota were composed of several predominant bacterial genera, including *Muribaculaceae, Lactobacillus,* and *UCG-010*. In the TPT exposure group, *o__Bacillales, f__Bacillaceae,* and *f__Caldicoprobacteraceae* abundance was relatively high, while *f__Oscillospiraceae, f__Fusobacteriaceae,* and *f__SC_I_84* abundance was relatively high in the control group. Gene function analyses in silkworm microbiota after TPT exposure showed that biosynthesis of ansamycins, fructose and mannose metabolism, glycerolipid metabolism, type II diabetes mellitus, glycolysis/gluconeogenesis, lipid metabolism, translation proteins, atrazine degradation, DNA repair and recombination proteins, nicotinate and nicotinamide metabolism were significantly increased. Collectively, our silkworm model identified gut microbial diversity risks and the adverse effects from TPT exposure, which were similar to other aquatic animals. Therefore, TPT levels in environmental samples must be monitored to prevent ecological harm.

## Introduction

Triphenyltin (TPT) is a synthetic chemical used as an antifouling paint in industry and a fungicide in agriculture^1^. When TPT is exposed to high temperatures and rain, it is released into water bodies^2,3^. From 2008, TPT-use was prohibited in antifouling paints as TPT concentrations in the environment and aquatic organisms can potentially harm human health^4^. In coastal areas of Hong Kong, China, it was previously reported that TPT concentrations were up to 3.8–11.7 ng/L in the upper seawater and 71.8–91.7 ng/g (w/w) in the sediment^5^.

It is accepted that TPT is an endocrine disruptor with high toxicity^6,7^. Many studies have reported its potential risks in aquatic organisms and other species and concluded that TPT impacts immunity, development, reproduction, and endocrine systems^7,8^. TPT disrupts lipid metabolism in *Marisa cornuarietis* at environmental concentrations^9^. TPT also interferes with retinal axon development in fish at low doses^10^, induces oxidative stress and apoptosis in zebrafish^11^, and disrupts glucocorticoid synthesis in the rat adrenal cortex via several mechanisms: 1) it lowers serine/threonine kinase 1 (AKT1) phosphorylation and Sirtuin 1 (SIRT1)/peroxisome proliferator-activated receptor-gamma coactivator 1α (PGC-1α) levels, 2) activates AMP-activated protein kinase (AMPK), and 3) possibly induces reactive oxidative species (ROS) production^12^. TPT also appears to affect reproduction and mortality at decreasing concentrations in temporally overlapping generations^13^.

As a representative lepidopteran insect vital for agricultural and forestry silk production, the silkworm (*Bombyx mori*) is an important model organism^14^. *Bombyx mori* has relatively weak resistance to stress and disease and is thus a promising biological model system for drug screening and antibiotic resistance and environmental pollutant toxicity studies^15–17^. In our previous study, we evaluated TPT toxicity using this model and reported that TPT exposure hindered ontogeny by affecting carbohydrate, lipid, and amino acid metabolism in the midgut^18^. Similarly, TPT toxic effects have been comprehensively investigated in multiple species, however, its effects on insect gut microbiota diversity remain obscure. In this study, 16S rDNA sequencing was used to examine the impact of TPT exposure on microbial diversity in the silkworm midgut. Our findings shed new light on how TPT exposure impacts gut microbial homeostasis and highlights TPT ecotoxicity risks in environment and aquatic organisms.

## Materials and methods

### Chemicals

TPT chloride (CAS 639-58-7 and > 96% purity) was obtained from Sigma Aldrich (St. Louis, MO, USA) and diluted in absolute ethanol to generate a 10 μg/mL stock solution.

### Silkworm feeding

*Bombyx mori* (Jingsong × Haoyue strain) were fed formula feed^18^ supplemented with 0 and 2 μg/kg TPT for the entire 5th instar larval stage. In briefly, all larvae were reared under a 12-h light/dark cycle at a constant temperature of 26 °C. The control group (90 larvae) and TPT-exposure group (90 larvae) contained 3 replicates respectively. The formula feed was prepared and cut into 4*4 cm. To avoid the loss of water in artificial feed, and the prepared feed were place in a sterilized plastic box. The control group and TPT-exposure group were as consistent as possible. The midguts of silkworms were collected for further investigation after 4 days of feeding.

### DNA extraction, amplification, and 16S rDNA sequencing

Gut content bacterial DNA was extracted using the QIAamp PowerFecal DNA Kit (Qiagen, Hilden, Germany), amplified using polymerase chain reaction targeting the V3–V4 hypervariable region of 16S rDNA, and then sequenced using Illumina NovaSeq6000 (Illumina Inc., San Diego, CA, USA). Raw 16S rDNA sequencing data were submitted to the Sequence Read Archive with the accession number PRJNA963166 (https://www.ncbi.nlm.nih.gov/bioproject/?term=PRJNA963166).

### Data preprocessing and statistical analysis

Trimmomatic (Version 0.35)^19^ and FLASH (Version 1.2.11)^20^ programs were used to remove low-quality reads and assemble paired-end reads, respectively. Clean reads were clustered into operational taxonomic units (OTUs) using Vsearch (Version 2.4.2)^21^. All valid reads were blasted against the Silva Version 138 database (16s/18s rDNA) using the RDP classifier (Version 2.2)^22^ and Unite database using BLAST^23^. The microbial diversity in the midgut content isolated from silkworm larvae was analyzed by the α-diversity, including species richness (Chao 1) and community diversity (Shannon, Simpson and PD whole tree). The Unifrac Principal coordinates analysis (PCoA) between the TPT exposure group and the control group was drawn with the QIIME software (version 1.8.0). To characterize the microbial differences between the different groups, linear discriminant analysis (LDA) effect size (LEfSe) analysis was employed using Galaxy online tools (http://huttenhower.sph.harvard.edu/lefse/).

Through the pathway enrichment analysis of differential metabolites, it is helpful to understand the mechanism of metabolic pathway change in different samples. Pathway enrichment analysis was performed using KEGG ID of differential metabolites, and the enrichment results of metabolic pathways were obtained. Hypergeometric tests were applied to identify pathway entries that were significantly enriched in significantly differentially expressed metabolites compared to the overall background. Its calculation formula is as follows:

$$ P = 1 - \sum\nolimits_{i = 0}^{m - 1} {\frac{{\left( {\begin{array}{*{20}c} M \\ i \\ \end{array} } \right)\left( {\begin{array}{*{20}c} {N - M} \\ {n - i} \\ \end{array} } \right)}}{{\left( {\begin{array}{*{20}c} N \\ n \\ \end{array} } \right)}}} $$

N is the total number of metabolites; n is the number of differentially expressed metabolites in N. M is the number of metabolites annotated in a particular pathway; m is the number of differential metabolites in a particular pathway. With *p*-value ≤ 0.05 as the threshold, the pathway that meets this condition is the pathway that is significantly enriched in differential metabolites. The smaller the *p*-value, the more significant the difference of the metabolic pathway.

## Results

### Data acquisition and analysis

**Figure 1:**
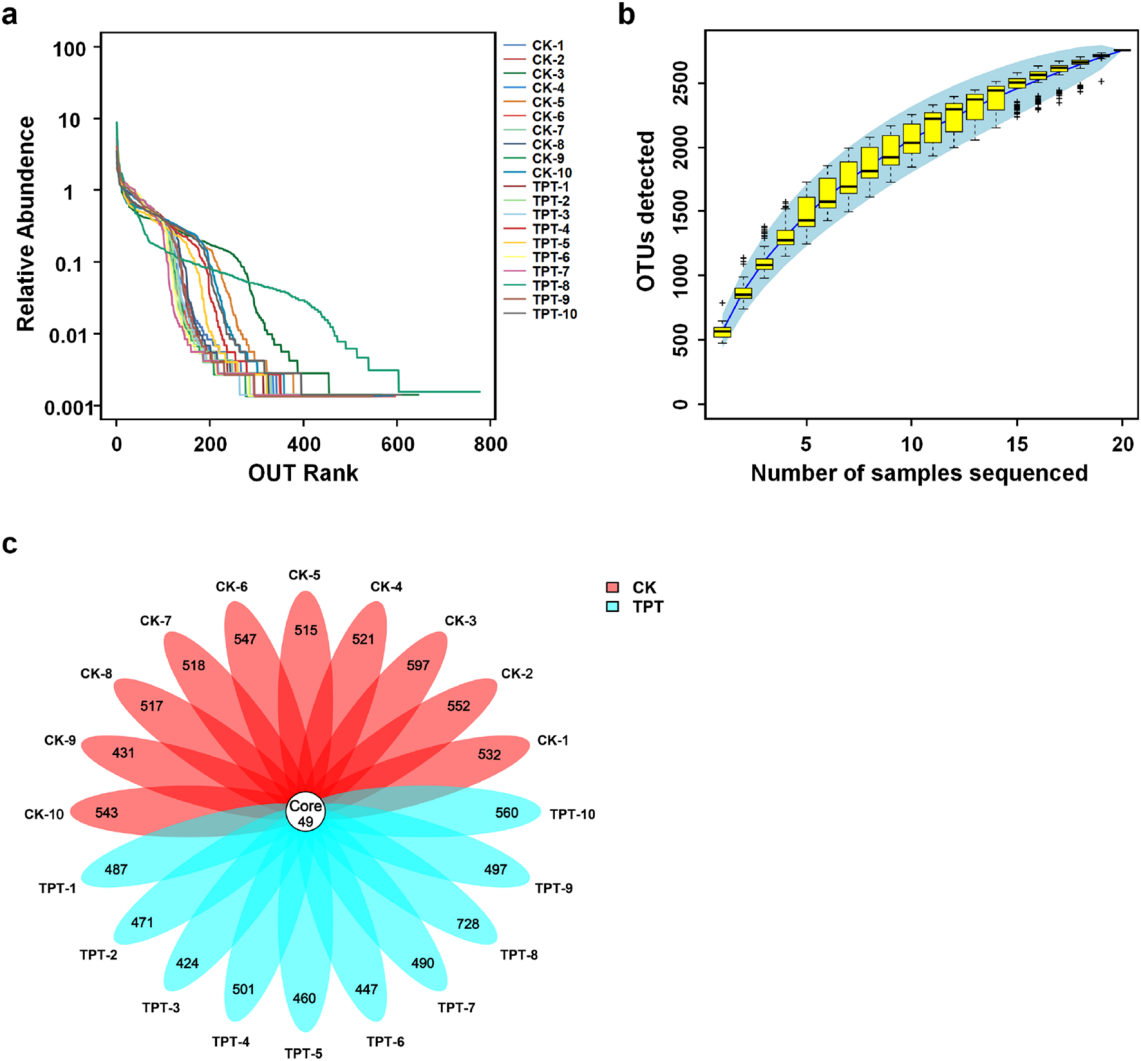
TPT exposure effects on microbial species and operational taxonomic units (OTUs). (**a**) Rarefaction and (**b**) rank abundance curves. (**c**) OTUs were identified between the control (CK) and TPT-exposure group (TPT) samples.

In total, 805,966 and 810,383 raw sequences were collected from the control (CK) and TPT exposure groups (TPT), respectively (Table S1). After quality testing, 765,471 and 763,686 high-quality reads were respectively acquired with an average of 76,458 (range 75,112–78,588) clean reads/sample. Rarefaction and rank abundance curve results indicated that nearly all microbial species were recognized (Fig. 1a,b). Using Vsearch (Version 2.4.2) software to classify OTUs based on nucleotide-sequence similarity, 5273 and 5065 OTUs were identified in CK and TPT-exposure group samples, respectively, ranging from 424 to 728 OTUs/sample (Fig. 1c).

### Comparative analyses of gut microbial diversity

**Figure 2:**
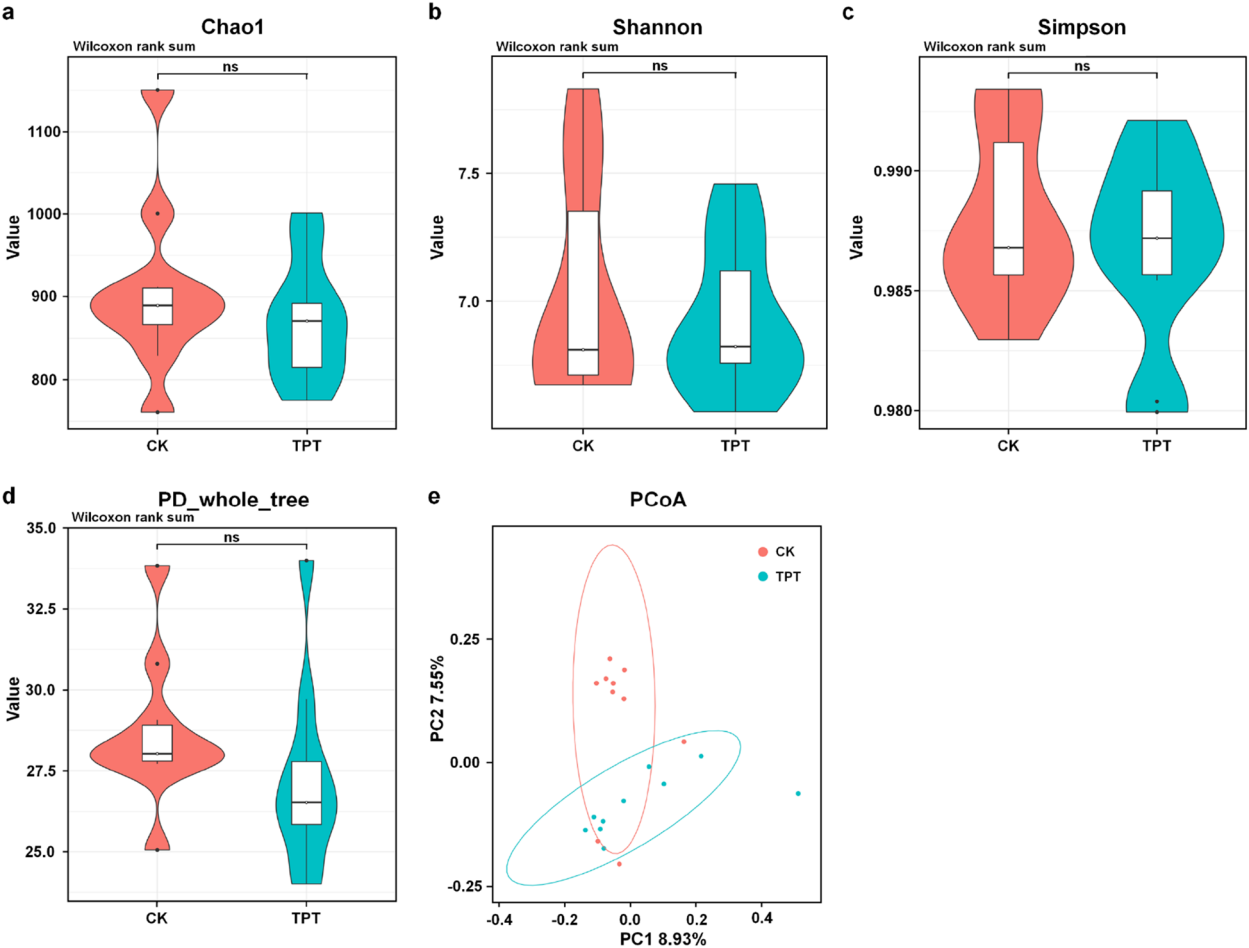
Comparative analyses of gut microbial diversity. Alpha diversity index analyses: (**a**) Chao1, (**b**) Simpson, (**c**) Shannon, and (**d**) PD_whole_tree indices. (**e**) Beta-diversity analysis using PCoA.

To further explore the effects of TPT exposure on silkworm midgut microbiota, alpha and beta diversity indices indicating microbial diversity were analyzed. Comparative analyses of diversity indices between two groups showed no significant differences in Chao1, Simpson, Shannon, and PD_whole_tree indices (Fig. 2a–d). Diversity index dilution curve showed that the curve will eventually flatten, indicating that the sequencing depth is large enough and the established libraries can truly and effectively reflect the diversity of bacterial in the samples with research significance and practical value (Fig. S1). Alpha-diversity analyses revealed that TPT exposure could induce the fluctuations of gut microbial diversity and abundance in silkworms.

Beta-diversity was analyzed using principal coordinate analysis (PCoA) (Fig. 2e), which reflected gut microbial similarities and differences between groups, and showed that most of individuals in both groups were separated and minority individuals were clustered together, suggesting that TPT exposure had an obvious impact on major gut microbiota components.

### Comparative analysis of microbial taxonomic composition

In total, 23 phyla and 353 genera were recognized in the CK group, while 20 phyla and 358 genera were recognized in the TPT group (Table S2). At the genus level, midgut microbiota were composed of several predominant bacterial genera, including *Muribaculaceae*, *Lactobacillus*, *UCG-010*, *Bacteroides*, *UCG-005*, *Clostridium_sensu_stricto_1*, *Rikenellaceae_RC9_gut_group*, *Escherichia-Shigella*, *Sphingomonas*, *Burkholderia-Caballeronia-Paraburkholderia*, *Lachnospiraceae_NK4A136_group*, *Alistipes*, *Prevotella*, *[Eubacterium]_coprostanoligenes_group*, and *Christensenellaceae_R-7_group*.

**Figure 3:**
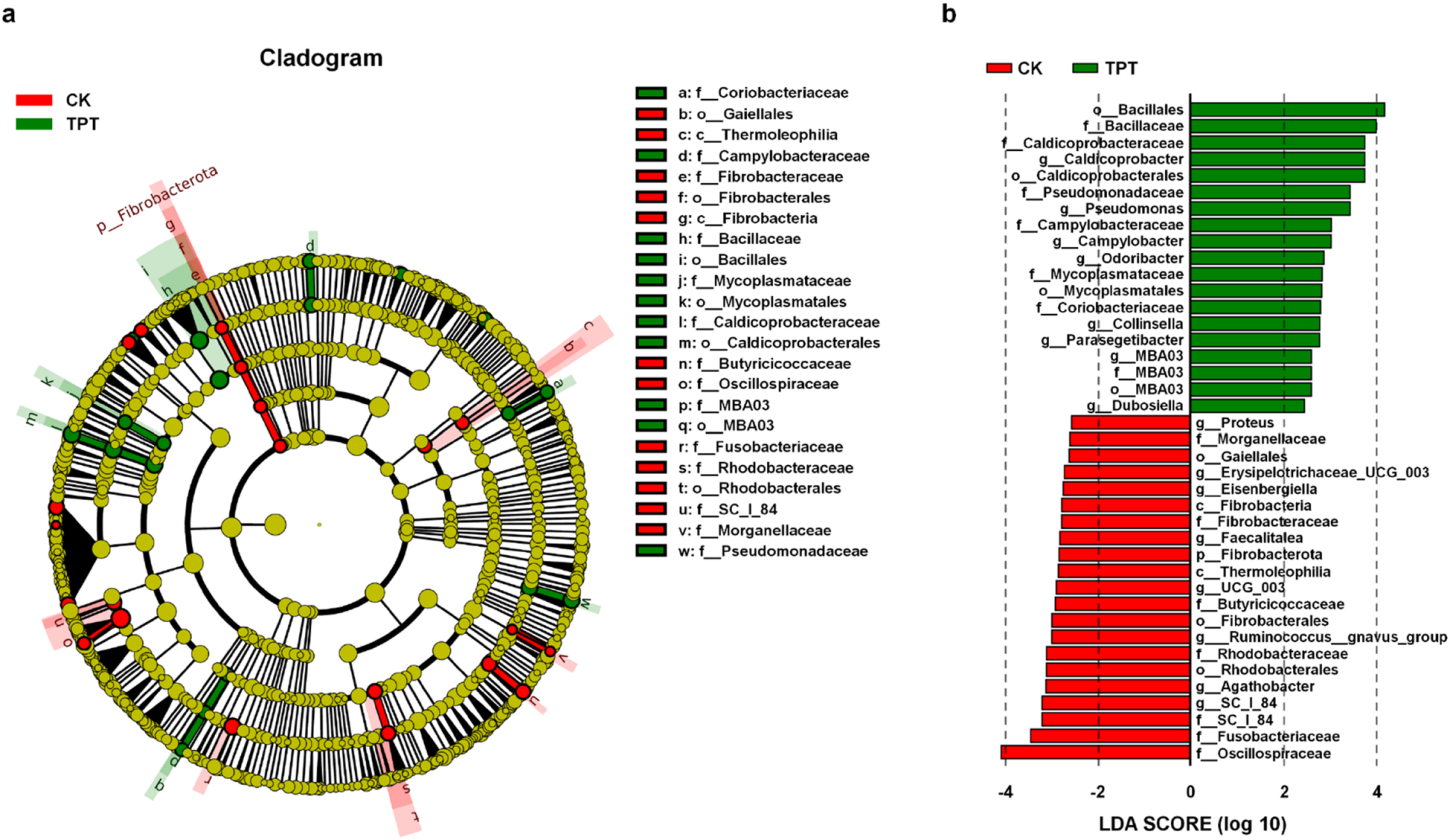
Comparative analysis of microbial taxonomic composition. (**a**) Annotation analysis of significantly different bacteria (SDB) based on the linear discriminant analysis coupled with effect size measurements (LEfSe). (**b**) Score plot of SDB based on LEfSe analysis. Green and red indicates a SDB that is more abundant in the TPT and CK group, respectively.

Biomarkers reflecting taxonomic levels between groups were analyzed using the linear discriminant analysis coupled with effect size measurements (LEfSe). Analysis results of significantly different bacteria show that *f__Coriobacteriaceae*, *f__Campylobacteraceae*, *f__Bacillaceae*, *o__Bacillales*, *f__Mycoplasmataceae*, *o__Mycoplasmatales*, *f__Caldicoprobacteraceae*, *o__Caldicoprobacterales*, *f__MBA03*, *o__MBA03*, and *f__Pseudomonadaceae* were more abundant in the TPT group; whereas *o__Gaiellales*, *c__Thermoleophilia*, *f__Fibrobacteraceae*, *o__Fibrobacterales*, *c__Fibrobacteria*, *f__Butyricicoccaceae*, *f__Oscillospiraceae*, *f__Fusobacteriaceae*, *f__Rhodobacteraceae*, *o__Rhodobacterales*, *f__SC_I_84*, and *f__Morganellaceae* were more abundant in the CK group (Fig. 3a,b, Tables S3, S4).

### Predicted functional potential of the altered microbiome

To explore altered genes in silkworm microbiota after TPT exposure, Kyoto Encyclopedia of Genes and Genomes^24,25^ (KEGG, www.KEGG.jp/KEGG/kegg1.html) orthology level 3 pathways were identified using PICRUSt2, and showed that biosynthesis of ansamycins, fructose and mannose metabolism, glycerolipid metabolism, type II diabetes mellitus, glycolysis/gluconeogenesis, lipid metabolism, translation proteins, atrazine degradation, DNA repair and recombination proteins, nicotinate and nicotinamide metabolism were significantly increased after TPT exposure (Fig. S2).

## Discussion

TPT is a toxic chemical used in various industrial and agricultural applications. TPT exposure risk studies have reported harmful effects on human health, including reproductive toxicity, immunotoxicity, neurotoxicity, and endocrine disruption^7,8^. TPT exposure occurs via inhalation, ingestion, or skin contact with contaminated water, soil, or food^26,27^. Therefore, proper protective measures should be taken to minimize TPT exposure and limit environmental contamination in occupational settings. In this study, we examined TPT exposure on silkworm microbial diversity and showed that TPT exposure induced the gut microbial diversity and abundance fluctuations (alpha-diversity index analyses). Similarly, beta-diversity analyses showed that TPT exposure had an obvious impact on major gut microbiota components.

It is reported that TPT exposure exerts a negative impact on microbial diversity, including bacteria, fungi, and algae^28–30^. Exposure alters microbial community structures, thereby reducing bacterial abundance and diversity while promoting pathogen growth. In our study, we found that TPT can upregulate the abundance of pathogen in the midgut, including f__Campylobacteraceae, f__Mycoplasmataceae, and o__Mycoplasmatales (Fig. 3). Members of the family Campylobacteraceae neither ferment nor oxidize carbohydrates, instead they obtain energy from amino acids, or tricarboxylic acid cycle intermediates^31^. Members of the family Mycoplasmataceae can cause cell damage through different mechanisms, such as obtaining lipids and cholesterol on the cell membrane, causing membrane damage, and releasing neurotoxins, phosphatases, and hydrogen peroxide^32,33^. Therefore, we infer that TPT can cause damage to midgut cells by upregulating pathogenic bacteria in the intestine.

It was reported that the physiological stress of TPT has a negative impact on many intestinal bacteria, significantly reducing microbial diversity^34^. Furthermore, TPT could cause lipid metabolism abnormalities by affecting intestinal microbiome^34^. Our investigation found that TPT induced a significant increase in fructose and mannose metabolism, glycerolipid metabolism, glycolysis/gluconeogenesis, lipid metabolism, and translation proteins in the midgut microbiome of silkworms (Fig. S2). These changes may be related to the abnormality of carbohydrate, lipid, and amino acid metabolism in the midgut of silkworms caused by TPT exposure^18^. In our study, we used the silkworm model to explore the effects of TPT exposure on gut microbial diversity and observed that adverse effects were similar to other aquatic animals^34^. Therefore, TPT levels must be monitored in environmental samples to prevent potential ecological harm.

### Supplementary Information

Supplementary Information.

## Data Availability

The datasets generated and/or analysed during the current study are available in the NCBI repository, accession number PRJNA963166.
