# Supplementary material for: Short-term triphenyltin exposure alters microbial homeostasis in the silkworm (Bombyx mori) midgut
Source: Sci Rep. 2023 Sep 13;13:15183. doi: 10.1038/s41598-023-41948-y (PMC10499869; doi:10.1038/s41598-023-41948-y)
Supplement: Supplementary file 1 — Supplementary Information. [file 41598_2023_41948_MOESM1_ESM.zip › Supplemental Materials/Supplemental Materials.docx]

**Supplemental Materials**

**Short-term triphenyltin exposure alters microbial homeostasis in the silkworm (Bombyx mori) midgut**

Wenlin Zhou^a,1,^*, Xing Zhang^b,1^, Xuedong Chen^a^, Xuehui Wu^a^, Aihong Ye^a^, Jinru Cao^a^, Xiaolong Hu^c^

^a^*Institute of Sericulture and Tea, Zhejiang Academy of Agricultural Sciences, Hangzhou 310021, China*

^b^*School of Chemistry and Life science, Suzhou University of Science and Technology, Suzhou 215009, China*

^c^*School of Biology & Basic Medical Science, Soochow University, Suzhou 215123, China*

^1^These authors contributed equally to this work.

*Corresponding author.

E-mail address: zhouwl@zaas.ac.cn (W. Zhou).


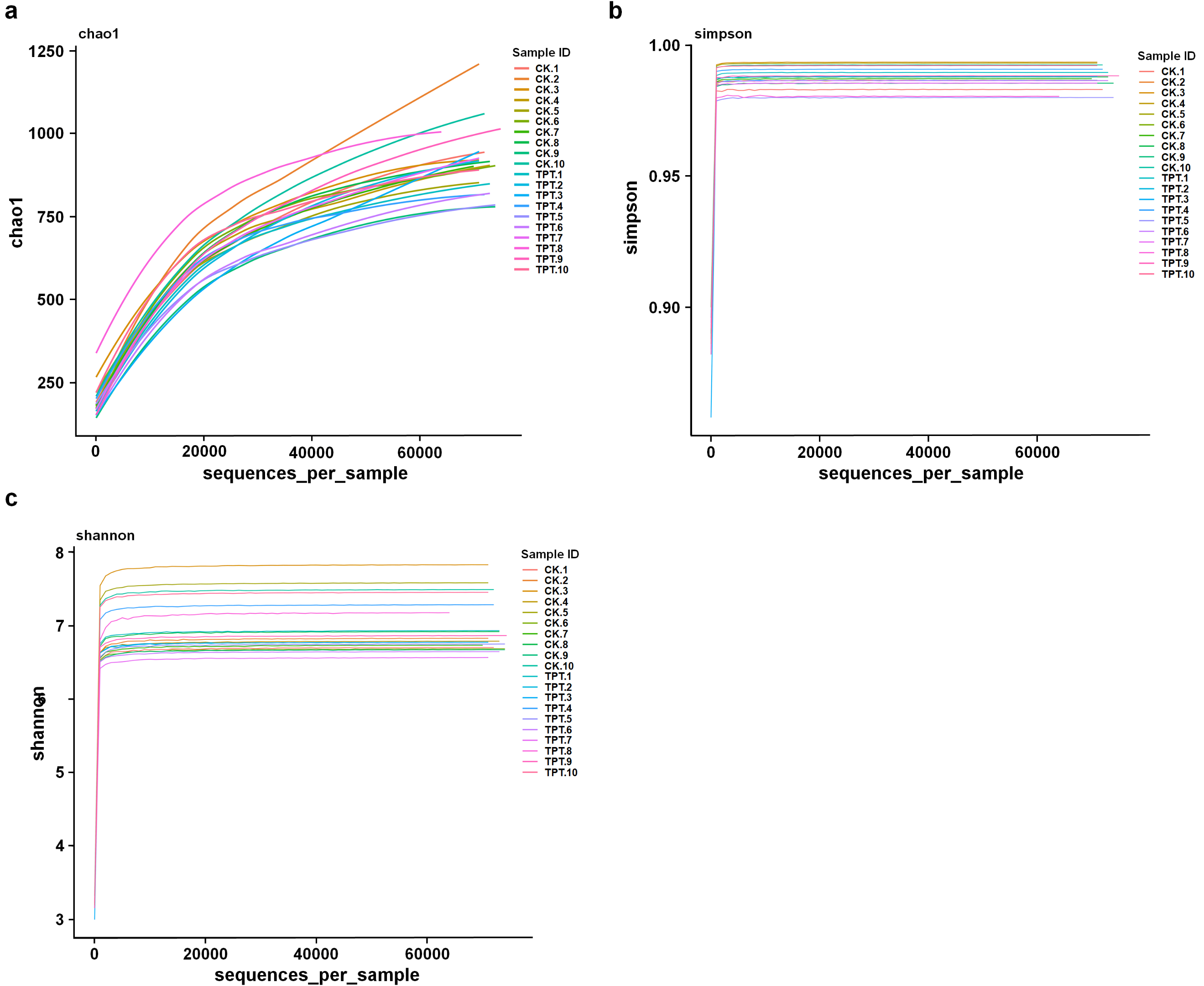


**Fig. S1.** Diversity index dilution curve. (a) Chao1, (b) Simpson, (c) Shannon. CK and TPT represent the control (CK) and TPT-exposure group (TPT) samples.


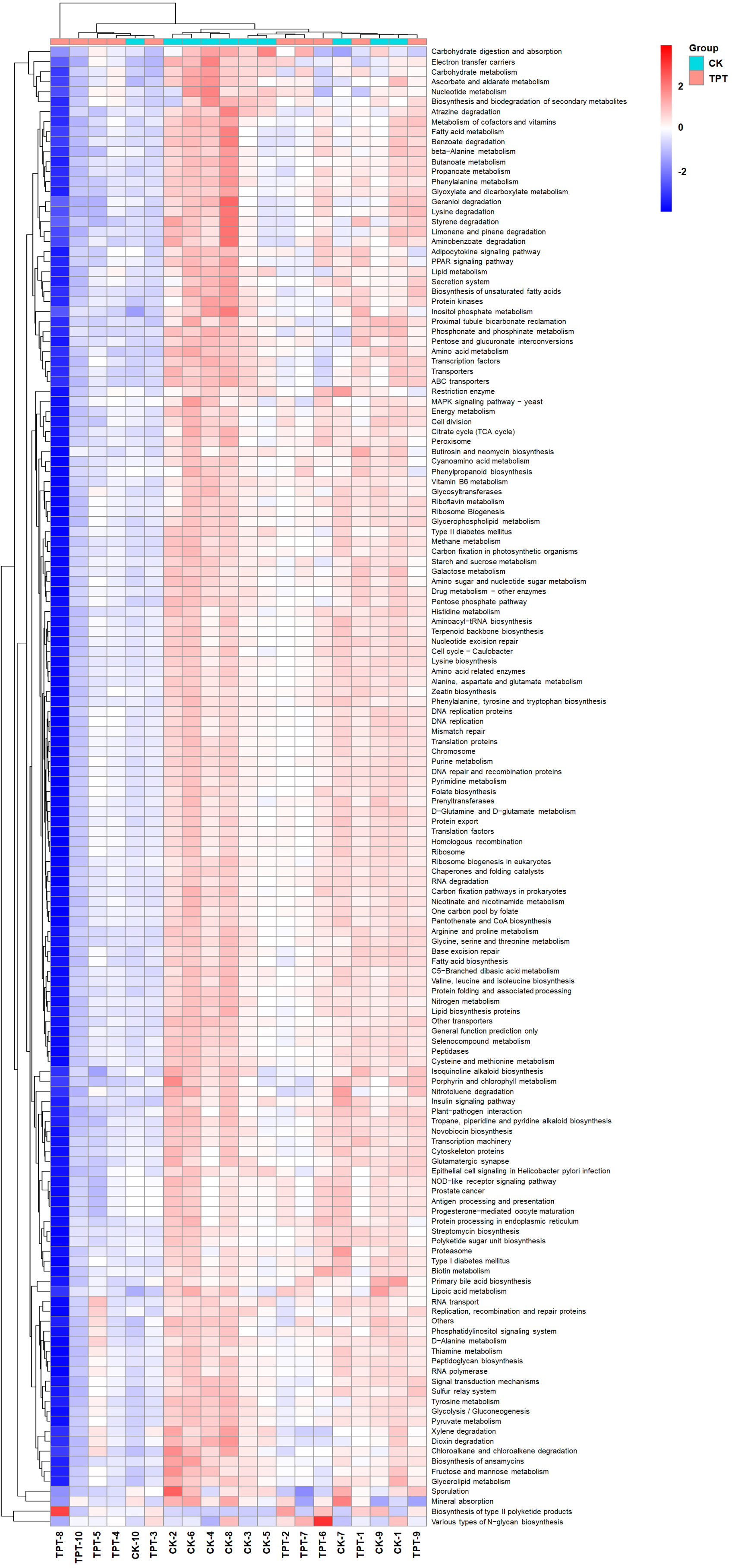


**Fig. S2.** Predicted functional potential of the altered microbiome. KEGG orthology level 3 pathways were shown using PICRUSt2.

**Table S1.** Sequencing data overview

| **Sample ID** | **clean_tags** | **valid_tags** | **valid_percent** | **valid minLength** | **valid meanLength** | **valid maxLength** | **OTU_counts** | **Total_OTUs** |
| --- | --- | --- | --- | --- | --- | --- | --- | --- |
| CK.1 | 75384 | 72560 | 96.25% | 255 | 406.31 | 442 | 581 | 2757 |
| CK.2 | 77124 | 71457 | 92.65% | 255 | 410.9 | 442 | 601 | 2757 |
| CK.3 | 75535 | 71378 | 94.50% | 252 | 406.09 | 442 | 646 | 2757 |
| CK.4 | 76874 | 73575 | 95.71% | 255 | 409.68 | 448 | 570 | 2757 |
| CK.5 | 76062 | 71864 | 94.48% | 255 | 402.78 | 453 | 564 | 2757 |
| CK.6 | 78364 | 74741 | 95.38% | 256 | 409.58 | 442 | 596 | 2757 |
| CK.7 | 75428 | 70426 | 93.37% | 256 | 408.87 | 442 | 567 | 2757 |
| CK.8 | 76516 | 73578 | 96.16% | 255 | 404.95 | 442 | 566 | 2757 |
| CK.9 | 76901 | 74605 | 97.01% | 253 | 409.32 | 442 | 480 | 2757 |
| CK.10 | 77283 | 72965 | 94.41% | 252 | 407.38 | 441 | 592 | 2757 |
| TPT.1 | 76488 | 73659 | 96.30% | 256 | 411.33 | 442 | 536 | 2757 |
| TPT.2 | 75191 | 71220 | 94.72% | 256 | 410.67 | 450 | 520 | 2757 |
| TPT.3 | 76256 | 71606 | 93.90% | 256 | 411.07 | 443 | 473 | 2757 |
| TPT.4 | 76485 | 72681 | 95.03% | 255 | 404.07 | 442 | 550 | 2757 |
| TPT.5 | 78588 | 74724 | 95.08% | 256 | 415.16 | 442 | 509 | 2757 |
| TPT.6 | 77204 | 73507 | 95.21% | 256 | 409.10 | 441 | 496 | 2757 |
| TPT.7 | 75254 | 71930 | 95.58% | 255 | 410.81 | 441 | 539 | 2757 |
| TPT.8 | 75728 | 64696 | 85.43% | 254 | 412.20 | 442 | 777 | 2757 |
| TPT.9 | 77380 | 75292 | 97.30% | 253 | 407.05 | 442 | 546 | 2757 |
| TPT.10 | 75112 | 71206 | 94.80% | 256 | 409.63 | 443 | 609 | 2757 |

CK = control group; TPT = TPT-exposure group.

**Table S2.** The abundance and annotation of OTUs in each sample at phylum and genus level.

**Table S3**. LEfSe analysis analysis between the control and TPT-exposure group.

**Table S4.** The relative abundance of differential gut microbes.
